# The “Diabetes Comorbidome”: A Different Way for Health Professionals to Approach the Comorbidity Burden of Diabetes

**DOI:** 10.3390/healthcare10081459

**Published:** 2022-08-03

**Authors:** Salvatore Corrao, Giuseppe Natoli, Alessandro Nobili, Pier Mannuccio Mannucci, Francesco Perticone, Vincenzo Arcoraci, Christiano Argano

**Affiliations:** 1 Internal Medicine Department IGR, National Relevance Hospital Trust, ARNAS Civico, Di Cristina e Benfratelli, 90127 Palermo, Italy; peppenatoli@gmail.com (G.N.); chargano@yahoo.it (C.A.); 2 Department of Health Promotion Sciences, Maternal and Infant Care, Internal Medicine and Medical Specialties (PROMISE), University of Palermo, 90127 Palermo, Italy; 3 Department of Health Policy, Istituto di Ricerche Farmacologiche Mario Negri IRCCS, 20156 Milan, Italy; alessandro.nobili@marionegri.it; 4 Scientific Direction, IRCCS Foundation Ca’ Granda Ospedale Maggiore Policlinico, 20122 Milan, Italy; piermannuccio.mannucci@policlinico.mi.it; 5 Department of Medical and Surgical Sciences, University Magna Graecia of Catanzaro, 88100 Catanzaro, Italy; perticone@unicz.it; 6 Department of Clinical and Experimental Medicine, University of Messina, 98122 Messina, Italy; vincenzo.arcoraci@unime.it

**Keywords:** “Diabetes Comorbidome”, comorbidities, dementia, cancer, in-hospital mortality, 3-month mortality, 1-year mortality

## Abstract

(1) Background: The disease burden related to diabetes is increasing greatly, particularly in older subjects. A more comprehensive approach towards the assessment and management of diabetes’ comorbidities is necessary. The aim of this study was to implement our previous data identifying and representing the prevalence of the comorbidities, their association with mortality, and the strength of their relationship in hospitalized elderly patients with diabetes, developing, at the same time, a new graphic representation model of the comorbidome called “Diabetes Comorbidome”. (2) Methods: Data were collected from the RePoSi register. Comorbidities, socio-demographic data, severity and comorbidity indexes (Cumulative Illness rating Scale CIRS-SI and CIRS-CI), and functional status (Barthel Index), were recorded. Mortality rates were assessed in hospital and 3 and 12 months after discharge. (3) Results: Of the 4714 hospitalized elderly patients, 1378 had diabetes. The comorbidities distribution showed that arterial hypertension (57.1%), ischemic heart disease (31.4%), chronic renal failure (28.8%), atrial fibrillation (25.6%), and COPD (22.7%), were the more frequent in subjects with diabetes. The graphic comorbidome showed that the strongest predictors of death at in hospital and at the 3-month follow-up were dementia and cancer. At the 1-year follow-up, cancer was the first comorbidity independently associated with mortality. (4) Conclusions: The “Diabetes Comorbidome” represents the perfect instrument for determining the prevalence of comorbidities and the strength of their relationship with risk of death, as well as the need for an effective treatment for improving clinical outcomes.

## 1. Introduction

Diabetes represents a major clinical and public health problem. According to the International Diabetes Federation, diabetes was estimated to affect 451 million adults globally in 2017, 1 in 11 adults aged 20 to 79 years [1], with a projected increase to 693 million by 2045 [2]. At this time, numerically, diabetes, if it were a nation, would be the third most populated country in the world [3]. The World Health Organization considers that diabetes will constitute the seventh leading cause of mortality worldwide in 2030. [4].

The burden of diabetes has increased significantly in recent decades and will continue to soar in the next few decades, especially in the older population. More than 20% of people aged 65 years and older have diabetes [5], and, among them, about 60% have at least one comorbidity [6], and up to 40% have four or more comorbidities [7]. 

Diabetes-related comorbidities are associated with a patient’s quality of life, health status, hospitalization, and outcomes [8,9,10]. It is worth outlining that, in 2017, disability-adjusted life-years (DALYs) associated with diabetes were 67.9 million, with a projection to 79.3 million in 2025. [11]. For this reason, some authors [6] have suggested a more comprehensive approach to the diagnosis, assessment, and management of diabetes and its comorbidities in older adults. In this sense, the utilization of a specific tool such as “comorbidome” could be helpful. The “comorbidome” is a graphic representation of the prevalence and the risk of death of comorbidities, similar to the solar system, developed by Divo and colleagues that identified the comorbidities related to an increased mortality in COPD [12]. 

According to the original design of Divo et al., the size of the circles expresses the prevalence of the single disease, while the distance to the center indicates the risk of death (the closer the comorbidity is to the center, the higher the risk of death). 

The importance of this graphic representation lies in the fact that it is possible to identify the most relevant and statistically important comorbidity. Recent studies have used comorbidome to represent associations between sex-specific comorbidities and mortality in COPD subjects [13], as well as comorbidities associated with higher risk of acute heart failure [14] Moreover, other approaches, such as a comorbidity network, have been used to develop a diabetes-risk prediction model [15]

In the light of the above reasons, the aim of this study was to investigate the prevalence of diabetes in the REPOSI population, focusing on comorbidities, and to study their association with mortality in-hospital, at a 3-month follow-up, and at a 1-year follow-up, utilizing, concomitantly, a new graphic representation model of comorbidome.

## 2. Materials and Methods

### 2.1. Data Collection and Study Population

Data concerning patients were extracted retrospectively from the frame of the RePoSI project. REPOSI is an independent and collaborative register, organized by the Italian Society of Internal Medicine (SIMI), the Fondazione Ca’ Granda Ospedale Maggiore Policlinico, and the Mario Negri Institute for Pharmacological Research. The introduction of the register was aimed at recruiting, monitoring, and evaluating older hospitalized patients aged 65 or over admitted to 102 Italian internal medicine and geriatric wards, with data collected every 2 years from 2008 onwards. The project’s design has been previously described in detail [16,17]. For this study, all 4713 patients recorded in the REPOSI Register between 2010 and 2016 were considered. All patient with and without diabetes were included in the present analysis. All patients provided informed consent. Data were collected in full compliance with the Italian law on personal data protection, and the REPOSI study was approved by the Ethics Committee of each participating center.

### 2.2. Socio-Demographic and Clinical Characteristics

Socio-demographic variables including age, sex, body mass index, and information about lifestyles were considered. The following clinical characteristics were evaluated: comorbidities and performance in activities of daily living at hospital admission (measured by means of the Barthel Index [BI] [18,19] and severity and comorbidity index (assessed by the Cumulative-Illness-Rating-Scale CIRS-s and CIRS-c, respectively) [20]. The association between comorbidities and in-hospital, 3-month, and 1-year mortality was analyzed.

### 2.3. Statistical Analysis

Stata Statistical Software2016, Release14 (Stata-Corp, College-Station, TX, USA) was used for database management and all analyses. Quantitative variables were summarized as mean (95% confidence intervals) and categorical variables as percentage. A Fisher’s exact-test for contingency tables, a z test, and a non-parametric Mann–Whitney-U-test were used when appropriate. Comorbidity distributions with a prevalence equal to or greater than 3% were reported.

#### The “Diabetes Comorbidome”

The “comorbidome” is the graphic representation of comorbidities and their association with mortality by Odds Ratio [OR]. The diameters of colored circles are functions of the prevalence of each comorbidity. The dotted-line circle represents the OR equal to 1, the outside area corresponds to OR < 1, and the inner area corresponds to OR > 1. All circles associated with a statistically significant increase in mortality are fully inside the dotted orbit. Mortality is set at the center (with the higher value = 4). The proximity to the center represents the stronger positive association with mortality. The comorbidities are represented graphically clockwise, from the first with the highest prevalence to the last with the lowest prevalence in patients with diabetes. The association with mortality was represented during hospitalization (panel A), at a three-month follow-up (panel B), and at a 1-year follow-up (panel C).

## 3. Results

As previously described [9], of 4714 hospitalized patients aged 65 years or older recorded in the REPOSI register during the years 2010–2016, 1378 subjects were affected by diabetes (29.2%).

**Figure 1:**
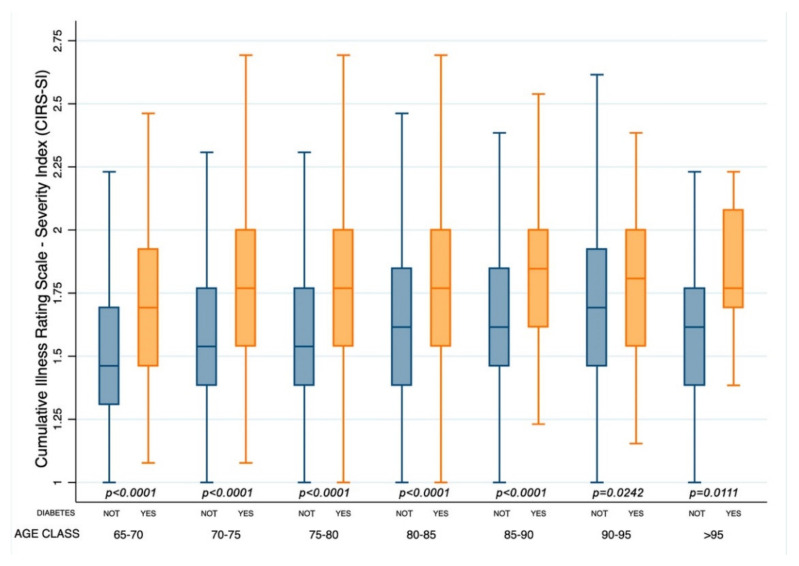
Box and whisker plot of the Cumulative Illness Rating Scale Severity Index [CIRS-SI] according to age classes in patients with or without diabetes (Yes or Not).

**Figure 2:**
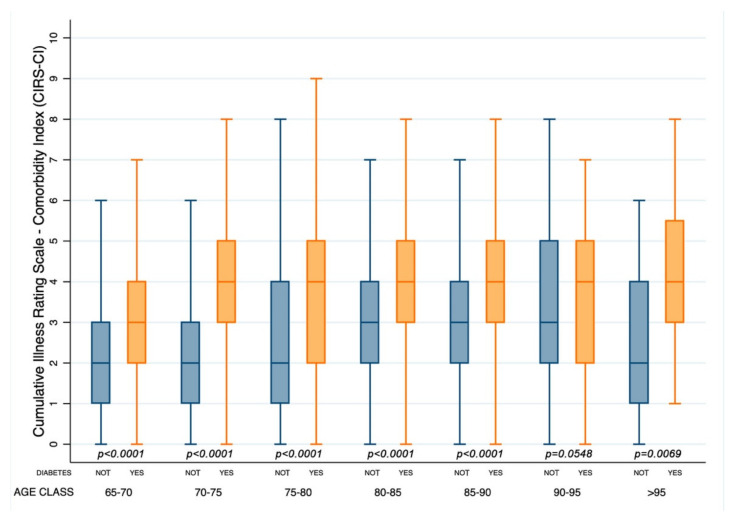
Box and whisker plot of the Cumulative Illness Rating Scale Comorbidity Index [CIRS-CI] according to age classes in patients with or without diabetes (Yes or Not).

Patients with diabetes had a significantly higher cumulative illness rating scale for the evaluation of severity and comorbidity index (1.80 (1.78–1.81) vs. 1.60 (1.59–1.61) and 3.81 (3.69–3.92) vs. 2.69 (2.62–2.75), respectively, (*p* < 0.0001)) and a lower BI score (76.7 (75,0–78.4) vs. 78.3 (77.1–79.4) (*p* = 0.0019)). Notably, a deeper novel analysis of CIRS-SI and CIRS-CI according to age groups showed that patients with diabetes had CIRS-SI and CIRS-CI scores significantly higher for all age classes (Figure 1 and Figure 2).

**Figure 3:**
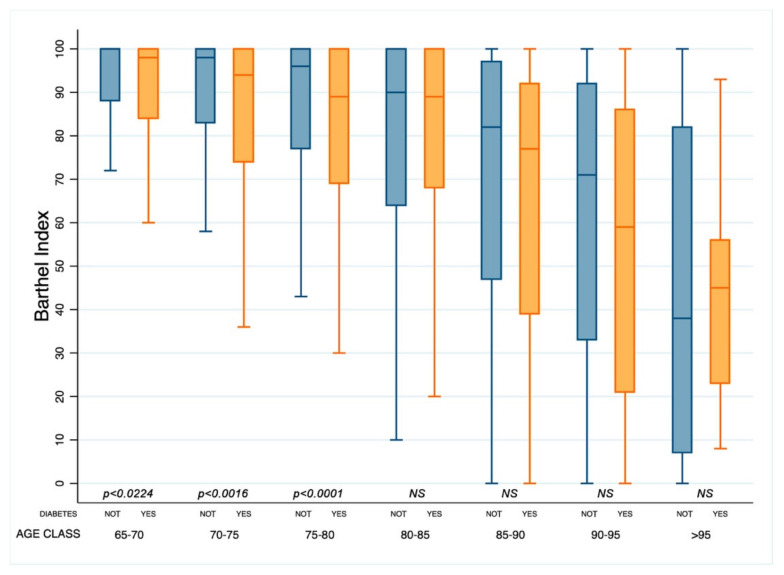
Box and whisker plot of the Barthel index according to age classes in patients with or without diabetes (Yes or Not). NS: not significant.

Moreover, subjects with diabetes between 65 and 80 years old had Barthel Index scores lower than people without diabetes (Figure 3).

**Figure 4:**
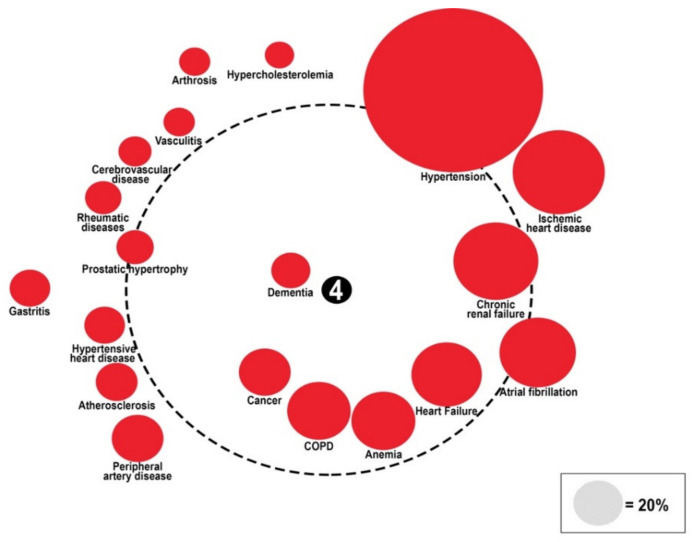
Comorbidome representation of in-hospital mortality in the RePoSI population with diabetes. Diameter of colored circles is function of the prevalence of each comorbidity. The dotted-line circle represents the OR equal to 1, the outside area corresponds to OR < 1, and the inner area correspond to OR > 1. All circles associated with a statistically significant increase in mortality are fully inside the dotted orbit. Mortality is set at the center (with the higher value = 4). The proximity to the center represents the stronger positive association with mortality. The comorbidities are represented graphically clockwise, from the first with the higher prevalence to the last with the lower prevalence in patients with diabetes.

**Figure 5:**
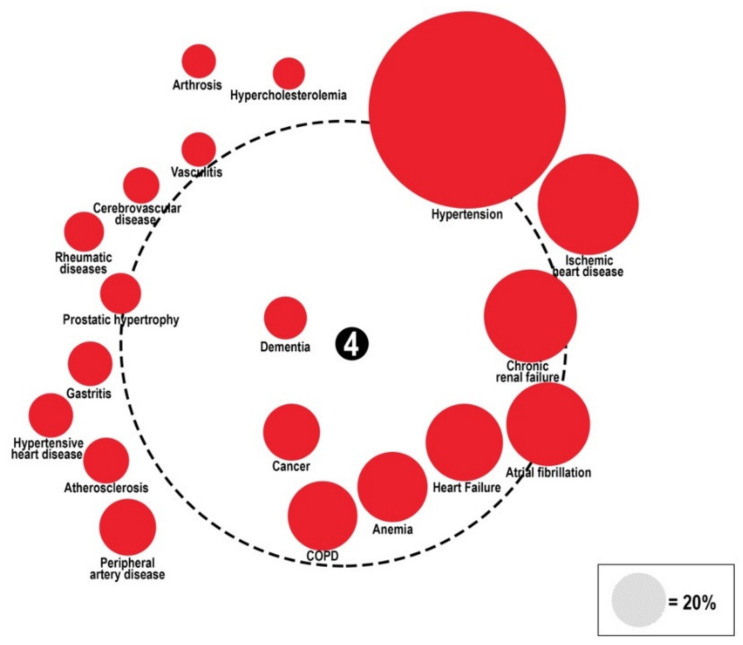
Comorbidome representation of 3-month mortality in the RePoSI population with diabetes. Diameter of colored circles is function of the prevalence of each comorbidity. The dotted-line circle represents the OR equal to 1, the outside area corresponds to OR < 1, and the inner area correspond to OR > 1. All circles associated with a statistically significant increase in mortality are fully inside the dotted orbit. Mortality is set at the center (with the higher value = 4). The proximity to the center represents the stronger positive association with mortality. The comorbidities are represented graphically clockwise, from the first with the higher prevalence to the last with the lower prevalence in patients with diabetes.

**Figure 6:**
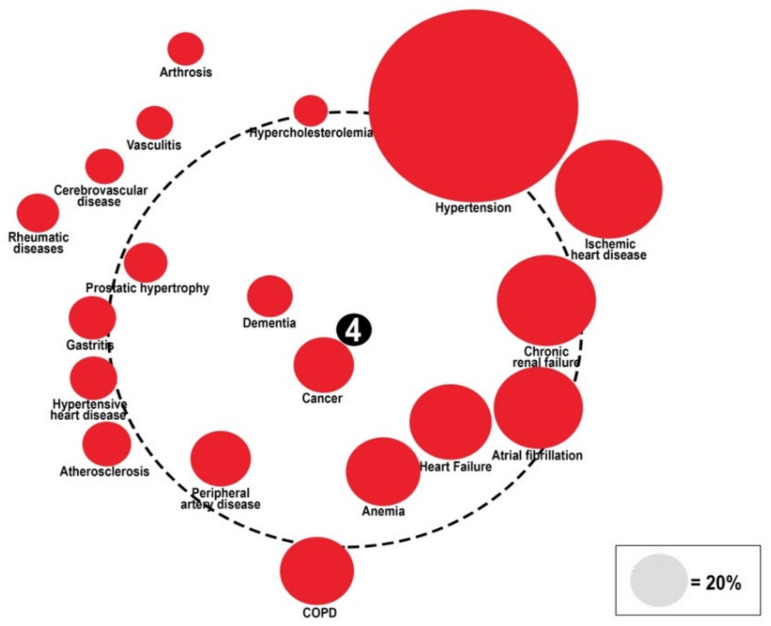
Comorbidome representation of 1-year mortality in the RePoSI population with diabetes. Diameter of colored circles is function of the prevalence of each comorbidity. The dotted-line circle represents the OR equal to 1, the outside area corresponds to OR < 1, and the inner area correspond to OR > 1. All circles associated with a statistically significant increase in mortality are fully inside the dotted orbit. Mortality is set at the center (with the higher value = 4). The proximity to the center represents the stronger positive association with mortality. The comorbidities are represented graphically clockwise, from the first with the higher prevalence to the last with the lower prevalence in patients with diabetes.

To evaluate the relationship between comorbidities and mortality during hospitalization and at follow-up, a graphic representation called the comorbidome was plotted (Figure 4, Figure 5 and Figure 6).

Hypertension was the comorbidity with the highest prevalence (57.1%), while heart failure (22.6%), anemia (22%), COPD (22.7%), cancer (17.5%), and dementia (9.1%) were the strongest predictors of mortality (OR > 1) in-hospital (OR 1.27, 1.11, 1.31, 1.74, 3.32, respectively) and at the 3-month follow-up (OR 1.36, 1.33, 1.12, 2.02, 2.39, respectively). At the 1-year follow-up, heart failure, anemia, cancer, and dementia, along with peripheral artery disease (17.3%), and prostatic hypertrophy (13.2%), were independently associated with mortality (OR 1.50, 1.29, 3.62, 2.10, 1.13, 1.14, respectively). See Appendix A.

## 4. Discussion

Despite the considerable clinical and economic burden of diabetes in older populations, clinical trials have historically excluded the oldest patients, particularly those with comorbidities [21]. For this reason, there is a need to have an effective approach to comorbidities to improve the care and outcomes for older patients with diabetes, especially at this time, where it the risk of hospitalization and death in patients with COVID-19 is always high [22]. The first important finding of our analysis regards the importance of the utilization of the CIRS assessment of comorbidities in the diabetic population.

Our data are consistent with a recent analysis that showed that the CIRS assessment of comorbidity burden represents the more useful clinical tool for the evaluation all-cause mortality in hospitalized elderly patients [23]. According to a recent study, CIRS-SI and CIRS-CI were higher in subjects with pneumonia than patients without pneumonia [24], and a CIRS index value >3 is significantly associated with gastrointestinal bleeding in elderly patients [25]. It is worth outlining the importance of CIRS as instrument of choice for multimorbidity assessment in clinical trials and its benefit to predict mortality, hospital readmission, and prolonged hospital stays [26,27]. Another important finding concerns the role of the Barthel index. A Barthel Index value ≤ 40, along with CIRS-SI and glycemia level ≥ 250 mg/dL, was the strongest predictor of in-hospital mortality for patients aged 65 years and older admitted in internal and geriatric wards [28].

According to recent studies, hyperglycemia was an independent predictor of the functional outcomes of ischemic patients measured by the Barthel index on admission [29].

In a longitudinal cohort study with a long follow-up of 11 years, the BI at admission to geriatric department is associated with short- and long-term mortality in both genders [30]. The most important innovation of our study is represented by the utilization of a novel modified data visualization: the “Diabetes comorbidome “.

The comorbidities are represented graphically clockwise, from the first with the highest prevalence to the last with the lowest prevalence.

The center represents mortality. The proximity to the center shows the strength of the association between the comorbidity and the risk of death. In our opinion, this is a simple tool that allows the researcher to understand, at a glance, the prevalence and impact of every single comorbidity. The utilization of the comorbidome, representative of the study of comorbidity patterns, may be useful in improving the clinical management of each specific subgroup of patients with a given index disease such as diabetes. Moreover, the knowledge of the possible physio-pathological interactions among comorbidities can contribute to the improvement of prevention and treatment strategies. As highlighted in different studies, the comorbidome is an effective tool to represent the comorbidities related to an increased mortality in COPD [12], the associations between sex specific comorbidities, and the mortality in COPD subjects [13], as well as comorbidities associated with higher risk of acute heart failure [14].

Our findings for the co-morbidity burden of diabetes are expected to be representative for a hospitalized-care setting and might be more useful for those taking strategic decision to reduce the length of hospital stays and unplanned readmission to hospital and create successful transitions from hospitals to local services or homes.

In our analysis heart failure, anemia, COPD, cancer, and dementia were strongly associated with in-hospital and 3-month mortality.

A possible explanation involved the role of a chronic low-grade inflammation state that is present in age-associated chronic conditions and, in particular, in diabetes [31].

This phenomenon is exacerbated by hospitalization, determining a progressive decline of cognitive and clinical status and quality of life [32,33]. Heart failure, anemia, COPD, cancer, and dementia str characterized by low-grade inflammation and are often associated with diabetes [12,34,35,36,37]. Evidence indicate that inflammation and, in particular, high levels of TNF-α, IL-β and IL-6 are factors involved in the development of type-1 diabetes [31,38], and TNF- α, IL-1, IL-6, IL-10, leptin, and adiponectin are the factors involved in the development of type-2 diabetes [31,39].

In addition, other studies showed the role of adiponectin and its relationship with cardiometabolic comorbidities [40,41,42,43] and the impact of chronic disease such as diabetes and cancer on the health-related quality of life in elderly population [33].

Although hypertension, ischemic heart disease, chronic renal failure, and atrial fibrillation are highly prevalent, the risk of death that these comorbidities give is not significant. In our opinion, the most likely reason is that these comorbidities are more often highlighted and treated by physicians, even if they are risk factors for the development of more lethal diseases such as heart failure and cardio-renal syndrome. Hypertension, particularly, is the most prevalent comorbidity, but there is no correlation with the risk of death; on the contrary, it has a protective role, particularly during hospitalization.

Our results are in agreement with previous studies that showed an inverse association between hypertension and mortality in older people [44,45].

At a 1-year follow-up, peripheral artery disease and prostatic hypertrophy were associated with mortality along with cancer, dementia, heart failure, and anemia.

Type-2 diabetes, along with obesity, is associated with prostatic hypertrophy [46]. Prostatic hypertrophy increases with age and may affect three out of four men in people aged 60 and older [47], and men with higher fasting glucose or with a diagnosis of diabetes may have a significant 3-fold and 2.3-fold higher risk of benign prostatic hypertrophy, respectively [48]. A likely explanation is that the diagnosis of prostatic hypertrophy in this subgroup of patients subsequently evolves into a cancer. In men with type-2 diabetes, benign prostatic hypertrophy could be a risk factor for bladder cancer [49].

Regarding the role of peripheral artery disease, it is well known that diabetes mellitus increases the incidence of peripheral artery disease, accelerates disease progression, and increases disease severity. Patients affected by peripheral artery disease and diabetes mellitus are at high risk for amputation. According to a recent meta-analysis, diabetes is associated with an increased risk of mortality in peripheral vascular disease, particularly in patients with critical limb ischemia [50]. Subjects with peripheral artery disease are at higher risk of cardiovascular events, particularly a 14.2% increase in risk for every percentage point increase in HbA1c, [51]. Dementia was strongly associated with mortality both in-hospital and at follow-up. Hanyu [52] identified a subgroup of patients with diabetes and dementia characterized by specific diabetes-related metabolic abnormalities such as advanced age, high hemoglobin A1c level, long course of diabetes, high frequency insulin treatment, low apolipoprotein E4 carrier, less-severe medial temporal lobe atrophy, impaired attention and executive function, less-impaired word recall, and slow progression of cognitive impairment. Moreover, a recent meta-analysis showed that diabetes increases the risk of cognitive impairment (cognitive impairment and dementia) by 1.25 to 1.91 times [53]. Our results are consistent with previous studies that showed dementia in type-2 diabetes is associated with an increasing risk of death during a follow-up of 12.7 ± 5.9 years [54]. Finally, cancer was associated with in-hospital and follow-up mortality. Particularly, it is the strongest comorbidity associated with the risk of mortality at 1-year follow-up. In this sense, our data are in agreement with previous studies, which showed a strongly association between diabetes and different malignancies such as breast cancer [55], pancreatic [56], liver [57], kidney [58], endometrial [59], colorectal [60], bladder [61] cancers, and non-Hodgkin’s lymphoma [62,63].

Hyperglycemia represents one of the key factors in the hypothesis that diabetes raises the cancer risk for both men and women [64,65,66]. Hyperglycemia can promote the cancer cells’ proliferation, invasion, and migration; it induces the apoptotic resistance and the metastatic effect and enhances the chemoresistance of tumor cells [67,68,69].

This study had some limitations. First, no specific information about diabetes duration is available. Second, HbA1c, the better indicator of chronic glycemic levels and risk for long-term complications, is lacking. Third, the REPOSI register was not specifically designed to evaluate clinical information. Fourth, our study population was different from the BODE cohort of Divo that included predominantly stable outpatients with COPD from pulmonary clinics with fewer comorbidities at initial recruitment.

Our population consisted of hospitalized elderly patients in internal medicine and geriatric wards with multiple and more severe diseases. The major strength of this study is the multicenter design of the REPOSI.

## 5. Conclusions

In summary, our analysis opens up new scenarios about the assessment of the burden of comorbidities that afflicted patients with diabetes through the identification of subsets of patients by comorbidities using the modified comorbidome as graphic representation. The most relevant aspect concerns the utilization of a tool such as the modified comorbidome as graphic representation of the prevalence of comorbidities and related outcomes. It could represent a perfect instrument for determining the importance of each comorbidity and the need of an effective treatment for the improvement of clinical outcomes. Our data should be considered by health authorities for the creation of discharge plans in agreement with local services in order to improve the quality of life and reduce mortality and the burden on the health system caused by re-hospitalizations. Further studies about complex and chronic diseases would use this graphic tool as an expression of the prevalence of comorbidities and the strength of their association with risk of death-related outcomes.

## Appendix A

**Investigators and co-authors of the REPOSI (REgistro POliterapie SIMI, Società Italiana di Medicina Interna).** 

***Steering Committee*****:** Pier Mannuccio Mannucci (*Chair*) (*Fondazione IRCCS Cà Granda Ospedale Maggiore Policlinico, Milano*), Alessandro Nobili (*co-chair*) (*Istituto di Ricerche Farmacologiche Mario Negri IRCCS, Milano*), Giorgio Sesti (*Presidente SIMI*), Antonello Pietrangelo (*Direttore CRIS—SIMI*), Francesco Perticone (*Università Magna Grecia Policlinico Mater Domini, Catanzaro*), Francesco Violi (*Policlinico Umberto I, Roma*), Gino Roberto Corazza, (*IRCCS Policlinico San Matteo di Pavia, Pavia*), Salvatore Corrao (*ARNAS Civico, Di Cristina, Benfratelli, DiBiMIS, Università di Palermo, Palermo*), Alessandra Marengoni (*Spedali Civili di Brescia, Brescia*), Francesco Salerno (*IRCCS Policlinico San Donato Milanese, Milano*), Matteo Cesari (*Fondazione Maugeri, Milano*), Mauro Tettamanti, Luca Pasina, Carlotta Franchi (*Istituto di Ricerche Farmacologiche Mario Negri IRCCS, Milano*).

***Clinical Data Monitoring and Revision*****:** Carlotta Franchi, Alessio Novella, Mauro Tettamanti, Gabriella Miglio (*Istituto di Ricerche Farmacologiche Mario Negri IRCCS, Milano*).

***Database Management and Statistics*****:** Mauro Tettamanti, Alessia Antonella Galbussera, Ilaria Ardoino, Alessio Novella (*Istituto di Ricerche Farmacologiche Mario Negri IRCCS, Milano*).

***Investigators*** **:**

- Domenico Prisco, Elena Silvestri, Giacomo Emmi, Alessandra Bettiol, Irene Mattioli (*Azienda Ospedaliero Universitaria Careggi Firenze, SOD Medicina Interna Interdisciplinare*);
- Gianni Biolo, Michela Zanetti, Giacomo Bartelloni, Michele Zaccari, Massimiliano Chiuch (*Azienda Sanitaria Universitaria Integrata di Trieste, Clinica Medica Generale e Terapia Medica*);
- Massimo Vanoli, Giulia Grignani, Edoardo Alessandro Pulixi (*Azienda Ospedaliera della Provincia di Lecco, Ospedale di Merate, Lecco, Medicina Interna*);
- Matteo Pirro, Graziana Lupattelli, Vanessa Bianconi, Riccardo Alcidi, Alessia Giotta, Massimo R. Mannarino (*Azienda Ospedaliera Santa Maria della Misericordia, Perugia, Medicina Interna, Angiologia Malattie da Arteriosclerosi*);
- Domenico Girelli, Fabiana Busti, Giacomo Marchi (*Azienda Ospedaliera Universitaria Integrata di Verona, Verona, Medicina Generale e Malattie Aterotrombotiche e Degenerative*);
- Mario Barbagallo, Ligia Dominguez, Vincenza Beneduce, Federica Cacioppo (*Azienda Ospedaliera Universitaria Policlinico Giaccone Policlinico di Palermo, Palermo, Unità Operativa di Geriatria e Lungodegenza*);
- Salvatore Corrao, Giuseppe Natoli, Salvatore Mularo, Massimo Raspanti, Christiano Argano (*A.R.N.A.S. Civico, Di Cristina, Benfratelli, Palermo, UOC Medicina Interna ad Indirizzo Geriatrico-Riabilitativo*);
- Marco Zoli, Maria Laura Matacena, Giuseppe Orio, Eleonora Magnolfi, Giovanni Serafini, Angelo Simili, Mattia Brunori, Ilaria Lazzari, Angelo Simili (*Azienda Ospedaliera Universitaria Policlinico S. Orsola-Malpighi, Bologna, Unità Operativa di Medicina Interna Zoli*);
- Maria Domenica Cappellini, Giovanna Fabio, Margherita Migone De Amicis, Giacomo De Luca, Natalia Scaramellini, Valeria Di Stefano, Simona Leoni, Sonia Seghezzi, Alessandra Danuto Di Mauro, Diletta Maira, Marta Mancarella (*Fondazione IRCCS Cà Granda Ospedale Maggiore Policlinico, Milano, Unità Operativa Medicina Interna IA*);
- Tiziano Lucchi, Paolo Dionigi Rossi, Marta Clerici, Simona Leoni, Alessandra Danuta Di Mauro, Giulia Bonini, Federica Conti, Silvia Prolo, Maddalena Fabrizi, Miriana Martelengo, Giulia Vigani (*Fondazione IRCCS Cà Granda Ospedale Maggiore Policlinico, Milano, Geriatria*);
- Antonio Di Sabatino, Emanuela Miceli, Marco Vincenzo Lenti, Martina Pisati, Costanza Caccia Dominioni, Lavinia Pitotti, Donatella Padula (*IRCCS Policlinico San Matteo di Pavia, Pavia, Clinica Medica I, Reparto 11*);
- Roberto Pontremoli, Valentina Beccati, Giulia Nobili, Giovanna Leoncini, Jacopo Alberto, Federico Cattaneo (*IRCCS Azienda Ospedaliera Universitaria San Martino-IST di Genova, Genova, Clinica di Medicina Interna 2*);
- Luigi Anastasio, Lucia Sofia, Maria Carbone (*Ospedale Civile Jazzolino di Vibo Valentia, Vibo Valentia, Medicina Generale*);
- Francesco Cipollone, Maria Teresa Guagnano, Ilaria Rossi, Emanuele Valeriani, Damiani D’Ardes, Lucia Esposito, Simona Sestili, Ermanno Angelucci (*Ospedale Clinicizzato SS.* *Annunziata, Chieti, Clinica Medica*);
- Gerardo Mancuso, Daniela Calipari, Mosè Bartone (*Ospedale Giovanni Paolo II Lamezia Terme, Catanzaro, Unità Operativa Complessa Medicina Interna*);
- Giuseppe Delitala, Maria Berria, Alessandro Delitala (*Azienda Ospedaliera—Universitaria di Sassari, Clinica Medica*);
- Maurizio Muscaritoli, Alessio Molfino, Enrico Petrillo, Antonella Giorgi, Christian Gracin, Giovanni Imbimbo (Policlinico Umberto I, Sapienza Università di Roma, Medicina Interna e Nutrizione Clinica Policlinico Umberto I);
- Giuseppe Zuccalà, Gabriella D’Aurizio (Policlinico Universitario A. Gemelli, Roma, Roma, Unità Operativa Complessa Medicina d’Urgenza e Pronto Soccorso);
- Giuseppe Romanelli, Alessandra Marengoni, Andrea Volpini, Daniela Lucente, Francesca Manzoni, Annalisa Pirozzi, Alberto Zucchelli (*Unità Operativa Complessa di Medicina I a indirizzo geriatrico, Spedali Civili, Montichiari, Brescia*);
- Antonio Picardi, Umberto Vespasiani Gentilucci, Paolo Gallo, Chiara Dell’Unto (*Università Campus Bio-Medico, Roma, Medicina Clinica-Epatologia*);
- Giuseppe Bellelli, Maurizio Corsi, Cesare Antonucci, Chiara Sidoli, Giulia Principato, Alessandra Bonfanti, Hajnalka Szabo, Paolo Mazzola, Andrea Piazzoli, Maurizio Corsi (*Università degli studi di Milano-Bicocca Ospedale S. Gerardo, Monza, Unità Operativa di Geriatria*);
- Franco Arturi, Elena Succurro, Bruno Tassone, Federica Giofrè (*Università degli Studi Magna Grecia, Policlinico Mater Domini, Catanzaro, Unità Operativa Complessa di Medicina Interna*);
- Maria Grazia Serra, Maria Antonietta Bleve (*Azienda Ospedaliera "Cardinale Panico" Tricase, Lecce, Unità Operativa Complessa Medicina*);
- Antonio Brucato, Teresa De Falco, Enrica Negro, Martino Brenna, Lucia Trotta, Giovanni Lorenzo Squintani (*ASST Fatebenefratelli—Sacco, Milano, Medicina Interna*);
- Maria Luisa Randi, Fabrizio Fabris, Irene Bertozzi, Giulia Bogoni, Maria Victoria Rabuini, Tancredi Prandini, Francesco Ratti, Chiara Zurlo, Lorenzo Cerruti, Elisabetta Cosi (*Azienda Ospedaliera Università di Padova, Padova, Clinica Medica I*);
- Roberto Manfredini, Fabio Fabbian, Benedetta Boari, Alfredo De Giorgi, Ruana Tiseo (*Azienda Ospedaliera—Universitaria Sant’Anna, Ferrara, Unità Operativa Clinica Medica*);
- Giuseppe Paolisso, Maria Rosaria Rizzo, Claudia Catalano, Irene Di Meo (*Azienda Ospedaliera Universitaria della Seconda Università degli Studi di Napoli, Napoli, VI Divisione di Medicina Interna e Malattie Nutrizionali dell’Invecchiamento*);
- Claudio Borghi, Enrico Strocchi, Eugenia Ianniello, Mario Soldati, Silvia Schiavone, Alessio Bragagni, Francesca Giulia Leoni, Valeria De Sando, Sara Scarduelli, Michela Cammarosano, Ilenia Pareo (*Azienda Ospedaliera Universitaria Policlinico S. Orsola-Malpighi, Bologna, Unità Operativa di Medicina Interna Borghi*);
- Carlo Sabbà, Francesco Saverio Vella, Patrizia Suppressa, Giovanni Michele De Vincenzo, Alessio Comitangelo, Emanuele Amoruso, Carlo Custodero, Giuseppe Re, Andrea Schilardi, Francesca Loparco (*Azienda Ospedaliero-Universitaria Consorziale Policlinico di Bari, Bari, Medicina Interna Universitaria C. Frugoni*);
- Luigi Fenoglio, Andrea Falcetta, Alessia Valentina Giraudo, Salvatore D’Aniano (*Azienda Sanitaria Ospedaliera Santa Croce e Carle di Cuneo, Cuneo, S. C. Medicina Interna*);
- Anna L. Fracanzani, Silvia Tiraboschi, Annalisa Cespiati, Giovanna Oberti, Giordano Sigon, Felice Cinque (*Fondazione IRCCS Cà Granda Ospedale Maggiore Policlinico, Milano, UOC Medicina Generale ad Indirizzo Metabolico*);
- Flora Peyvandi, Raffaella Rossio, Giulia Colombo, Pasquale Agosti, Erica Pagliaro (*Fondazione IRCCS Cà Granda Ospedale Maggiore Policlinico, Milano, Medicina Interna 2, Ematologia non tumorale e Coagulopatie*);
- Canetta Ciro, Valter Monzani, Valeria Savojardo, Giuliana Ceriani, Christian Folli (*Fondazione IRCCS Cà Granda Ospedale Maggiore Policlinico, Milano, Medicina Interna Alta Intensità di Cure*);
- Francesco Salerno, Giada Pallini (*IRCCS Policlinico San Donato e Università di Milano, San Donato Milanese, Medicina Interna*);
- Fabrizio Montecucco, Luciano Ottonello, Lara Caserza, Giulia Vischi, Salam Kassem, Luca Liberale (*IRCCS Ospedale Policlinico San Martino e Università di Genova, Genova, Clinica Medica 1, Medicina Interna e Specialità Mediche*);
- Nicola Lucio Liberato, Tiziana Tognin (*ASST di Pavia, UOSD Medicina Interna, Ospedale di Casorate Primo, Pavia*);
- Francesco Purrello, Antonino Di Pino, Salvatore Piro (*Ospedale Garibaldi Nesima, Catania, Unità Operativa Complessa di Medicina Interna*);
- Renzo Rozzini, Lina Falanga, Maria Stella Pisciotta, Francesco Baffa Bellucci, Stefano Buffelli, Camillo Ferrandina, Francesca Mazzeo, Elena Spazzini, Giulia Cono, Giulia Cesaroni (*Ospedale Poliambulanza, Brescia, Medicina Interna e Geriatria*);
- Giuseppe Montrucchio, Paolo Peasso, Edoardo Favale, Cesare Poletto, Carl Margaria, Maura Sanino (*Dipartimento di Scienze Mediche, Università di Torino, Città della Scienza e della Salute, Torino, Medicina Interna 2 Unità Indirizzo d’Urgenza*);
- Francesco Violi, Ludovica Perri (*Policlinico Umberto I, Roma, Prima Clinica Medica*);
- Luigina Guasti, Francesca Rotunno, Luana Castiglioni, Andrea Maresca, Alessandro Squizzato, Leonardo Campiotti, Alessandra Grossi, Roberto Davide Diprizio, Francesco Dentali (*Università degli Studi dell’Insubria, Ospedale di Circolo e Fondazione Macchi, Varese, Medicina e Geriatria*);
- Marco Bertolotti, Chiara Mussi, Giulia Lancellotti, Maria Vittoria Libbra, Matteo Galassi, Yasmine Grassi, Alessio Greco, Elena Bigi, Elisa Pellegrini, Laura Orlandi, Giulia Dondi, Lucia Carulli (*Università di Modena e Reggio Emilia, Azienda Ospedaliero-Universitaria di Modena; Ospedale Civile di Baggiovara, Unità Operativa di Geriatria*);
- Angela Sciacqua, Maria Perticone, Rosa Battaglia, Raffaele Maio, Aleandra Scozzafava, Valentino Condoleo, Tania Falbo, Lidia Colangelo; Marco Filice, Elvira Clausi (*Università Magna Grecia Policlinico Mater Domini, Catanzaro, Unità Operativa Malattie Cardiovascolari Geriatriche*);
- Vincenzo Stanghellini, Eugenio Ruggeri, Sara del Vecchio, Ilaria Benzoni (*Dipartimento di Scienze Mediche e Chirurgiche, Unità Operativa di Medicina Interna, Università degli Studi di Bologna/Azienda Ospedaliero—Universitaria S.Orsola-Malpighi, Bologna*);
- Andrea Salvi, Roberto Leonardi, Giampaolo Damiani (*Spedali Civili di Brescia, U.O. 3a Medicina Generale*);
- Gianluca Moroncini, William Capeci, Massimo Mattioli, Giuseppe Pio Martino, Lorenzo Biondi, Pietro Pettinari, Monica Ormas, Emanuele Filippini, Devis Benfaremo, Roberto Romiti (*Clinica Medica, Azienda Ospedaliera Universitaria**—Ospedali Riuniti di Ancona*);
- Riccardo Ghio, Anna Dal Col (*Azienda Ospedaliera Università San Martino, Genova, Medicina III*);
- Salvatore Minisola, Luciano Colangelo, Mirella Cilli, Giancarlo Labbadia (*Policlinico Umberto I, Roma, SMSC03—Medicina Interna F e Malattie Metaboliche dell’osso*);
- Antonella Afeltra, Benedetta Marigliano, Maria Elena Pipita (*Policlinico Campus Biomedico Roma, Roma, Medicina Clinica*);
- Pietro Castellino, Luca Zanoli, Alfio Gennaro, Agostino Gaudio, Samuele Pignataro (*Azienda Ospedaliera Universitaria Policlinico—V. Emanuele, Catania, Dipartimento di Medicina);*
- *Francesca Mete, Miriam Gino (Ospedale degli Infermi di Rivoli, Torino, Medicina Interna*);
- Guido Moreo, Silvia Prolo, Gloria Pina (*Clinica San Carlo Casa di Cura Polispecialistica, Paderno Dugnano, Milano, Unità Operativa di Medicina Generale Emilio Bernardelli*);
- Alberto Ballestrero, Fabio Ferrando, Roberta Gonella, Domenico Cerminara, Paolo Setti, Chiara Traversa, Camilla Scarsi (*Clinica Di Medicina Interna ad Indirizzo Oncologico, Azienda Ospedaliera Università San Martino di Genova*);
- Bruno Graziella, Stefano Baldassarre, Salvatore Fragapani, Gabriella Gruden (*Medicina Interna III, Ospedale S. Giovanni Battista Molinette, Torino*);
- Franco Berti, Giuseppe Famularo, Patrizia Tarsitani (*Azienda Ospedaliera San Camillo Forlanini, Roma, Medicina Interna II*);
- Roberto Castello, Michela Pasino (*Ospedale Civile Maggiore Borgo Trento, Verona, Medicina Generale e Sezione di Decisione Clinica*);
- Marcello Giuseppe Maggio Gian Paolo Ceda, Simonetta Morganti, Andrea Artoni, Margherita Grossi (*Azienda Ospedaliero Universitaria di Parma, U.O.C Clinica Geriatrica*);
- Stefano Del Giacco, Davide Firinu, Giulia Costanzo, Giacomo Argiolas, Giovanni Paoletti, Francesca Losa (*Policlinico Universitario Duilio Casula, Azienda Ospedaliero-Universitaria di Cagliari, Cagliari, Medicina Interna, Allergologia ed Immunologia Clinica*);
- Giuseppe Montalto, Anna Licata, Filippo Alessandro Montalto (*Azienda Ospedaliera Universitaria Policlinico Paolo Giaccone, Palermo, UOC di Medicina Interna*);
- Francesco Corica, Giorgio Basile, Antonino Catalano, Federica Bellone, Concetto Principato (*Azienda Ospedaliera Universitaria Policlinico G. Martino, Messina, Unità Operativa di Geriatria*);
- Lorenzo Malatino, Benedetta Stancanelli, Valentina Terranova, Salvatore Di Marca, Rosario Di Quattro, Lara La Malfa, Rossella Caruso (*Azienda Ospedaliera per l’Emergenza Cannizzaro, Catania, Clinica Medica Università di Catania*);
- Patrizia Mecocci, Carmelinda Ruggiero, Virginia Boccardi (*Università degli Studi di Perugia-Azienda Ospedaliera S.M. della Misericordia, Perugia, Struttura Complessa di Geriatria*);
- Tiziana Meschi, Andrea Ticinesi, Antonio Nouvenne (*Azienda Ospedaliera Universitaria di Parma, U.O Medicina Interna e Lungodegenza Critica*);
- Pietro Minuz, Luigi Fondrieschi, Giandomenico Nigro Imperiale, Sarah Morellini (*Azienda Ospedaliera Universitaria Verona, Policlinico GB Rossi, Verona, Medicina Generale per lo Studio ed il Trattamento dell’Ipertensione Arteriosa*);
- Mario Pirisi, Gian Paolo Fra, Daniele Sola, Mattia Bellan (*Azienda Ospedaliera Universitaria Maggiore della Carità, Medicina Interna 1*);
- Roberto Quadri, Erica Larovere, Marco Novelli (*Ospedale di Ciriè, ASL TO4, Torino, S.C. Medicina Interna*);
- Emilio Simeone, Rosa Scurti, Fabio Tolloso (*Ospedale Spirito Santo di Pescara, Geriatria*);
- Roberto Tarquini, Alice Valoriani, Silvia Dolenti, Giulia Vannini (*Ospedale San Giuseppe, Empoli**, USL Toscana Centro, Firenze, Medicina Interna I*);
- Riccardo Volpi, Pietro Bocchi, Alessandro Vignali (*Azienda Ospedaliera Universitaria di Parma, Clinica e Terapia Medica*);
- Sergio Harari, Chiara Lonati, Federico Napoli, Italia Aiello (*Divisione di Medicina Interna, Multimedica IRCSS, Milano*);
- Francesco Purrello, Antonino Di Pino (*Ospedale Garibaldi**—**Nesima—Catania, U.O.C Medicina Interna*);
- Teresa Salvatore, Lucio Monaco, Carmen Ricozzi (*Policlinico Università della Campania L. Vanvitelli, UOC Medicina Interna*);
- Alberto Pilotto, Ilaria Indiano, Federica Gandolfo (*Ente Ospedaliero Ospedali Galliera Genova, SC Geriatria Dipartimento Cure Geriatriche, Ortogeriatria e Riabilitazione*);
- Franco Laghi Pasini, Pier Leopoldo Capecchi (*Azienda Ospedaliera Universitaria Senese, Siena, Unità Operativa Complessa Medicina 2*);
- Ranuccio Nuti, Roberto Valenti, Martina Ruvio, Silvia Cappelli, Alberto Palazzuoli (*Azienda Ospedaliera Università Senese, Siena, Medicina Interna I*);
- Mauro Bernardi, Silvia Li Bassi, Luca Santi, Giacomo Zaccherini (*Azienda Ospedaliera Policlinico Sant’Orsola-Malpighi, Bologna, Semeiotica Medica Bernardi*);
- Vittorio Durante, Daniela Tirotta, Giovanna Eusebi (*Ospedale di Cattolica, Rimini, Medicina Interna*);
- Marco Cattaneo, Maria Valentina Amoruso, Paola Fracasso, Cristina Fasolino (*Azienda ospedaliera San Paolo, Milano, Medicina III*);
- Moreno Tresoldi, Enrica Bozzolo, Sarah Damanti (*IRCCS Ospedale San Raffaele—Milano, Medicina Generale e delle Cure Avanzate*);
- Massimo Porta, Miriam Gino (*AOU Città della Salute e della Scienza di Torino—Torino, Medicina Interna 1U*).

**Table A1:** Univariate analysis.

Variables	Inpatient with Diabetes (%)	O.R. In-Hospital Mortality	O.R. 3-Months Mortality	O.R. 12-Months Mortality
Hypertension	57.1	**0.53 (0.33–0.84)**	**0.60 (0.44–0.99)**	0.67 (0.49–1.11)
Ischemic heart disease	31.3	**0.55 (0.31–0.97)**	0.71 (0.54–1.43)	0.64 (0.26–1.49)
Chronic renal failure	28.9	1.19 (0.73–1.95)	1.17 (0.72–1.87)	1.13 (0.41–2.23)
Atrial fibrillation	26.0	0.92 (0.54–1.57)	1.07 (0.74–1.96)	1.28 (1.05–5.07)
COPD	22.7	1.31 (0.77–2.23)	1.12 (0.58–1.71)	0.79 (0.04–1.01)
Heart Failure	22.6	1.27 (0.77–2.13)	1.36 (0.81–2.20)	1.50 (0.85–4.38)
Anemia	22.0	1.11 (0.64–1.92)	1.33 (0.83–2.32)	1.29 (0.54–3.21)
Peripheral artery disease	17.6	0.61 (0.30–1.26)	**0.53 (0.22–0.98)**	1.13 (0.54–2.87)
Cancer	17.5	**1.74 (1.01–3.04)**	**2.02 (1.28–3.52)**	**3.62 (1.49–9.78)**
Atherosclerosis	14.2	0.74 (0.33–1.66)	0.60 (0.19–1.27)	0.78 (0.31–3.13)
Hypertensive heart disease	13.9	0.81 (0.40–1.65)	**0.36 (0.03–0.50)**	0.88 (0.37–1.13)
Gastritis	13.8	**0.17 (0.42–0.72)**	0.78 (0.34–2.07)	0.87 (0.19–2.43)
Prostatic hypertrophy	12.7	1.02 (0.52–2.03)	0.96 (0.52–1.89)	1.14 (0.70–4.78)
Rheumatic diseases	12.4	0.59 (0.23–1.51)	**0.40 (0.09–0.98)**	**0.32 (0.13–1.56)**
Cerebrovascular disease	11.3	0.59 (0.07–4.64)	0.89 (0.38–2.94)	0.57 (0.21–2.09)
Vasculitis	10.7	0.62 (0.15–2.69)	0.96 (0.48–2.60)	0.54 (0.23–2.34)
Arthrosis	10.6	0.33 (0.10–1.06)	**0.26 (0.08–0.79)**	**0.23 (0.14–0.78)**
Hypercholesterolemia	10.0	0.47 (0.17–1.32)	0.62 (0.34–1.70)	0.94 (0.33–4.77)
Dementia	9.1	**3.32 (1.95–5.66)**	**2.39 (1.01–2.83)**	**2.10 (1.03–3.53)**

Data statistically significant (*p* < 0.05) were reported in bold.
